# Supplementary material for: Stomatal CO2 responses at sub- and above-ambient CO2 levels employ different pathways in Arabidopsis
Source: Plant Physiol. 2024 Jun 4;196(1):608–20. doi: 10.1093/plphys/kiae320 (PMC11376393; doi:10.1093/plphys/kiae320)
Supplement: kiae320_Supplementary_Data [file kiae320_supplementary_data.zip › Supplementary Data.pdf]

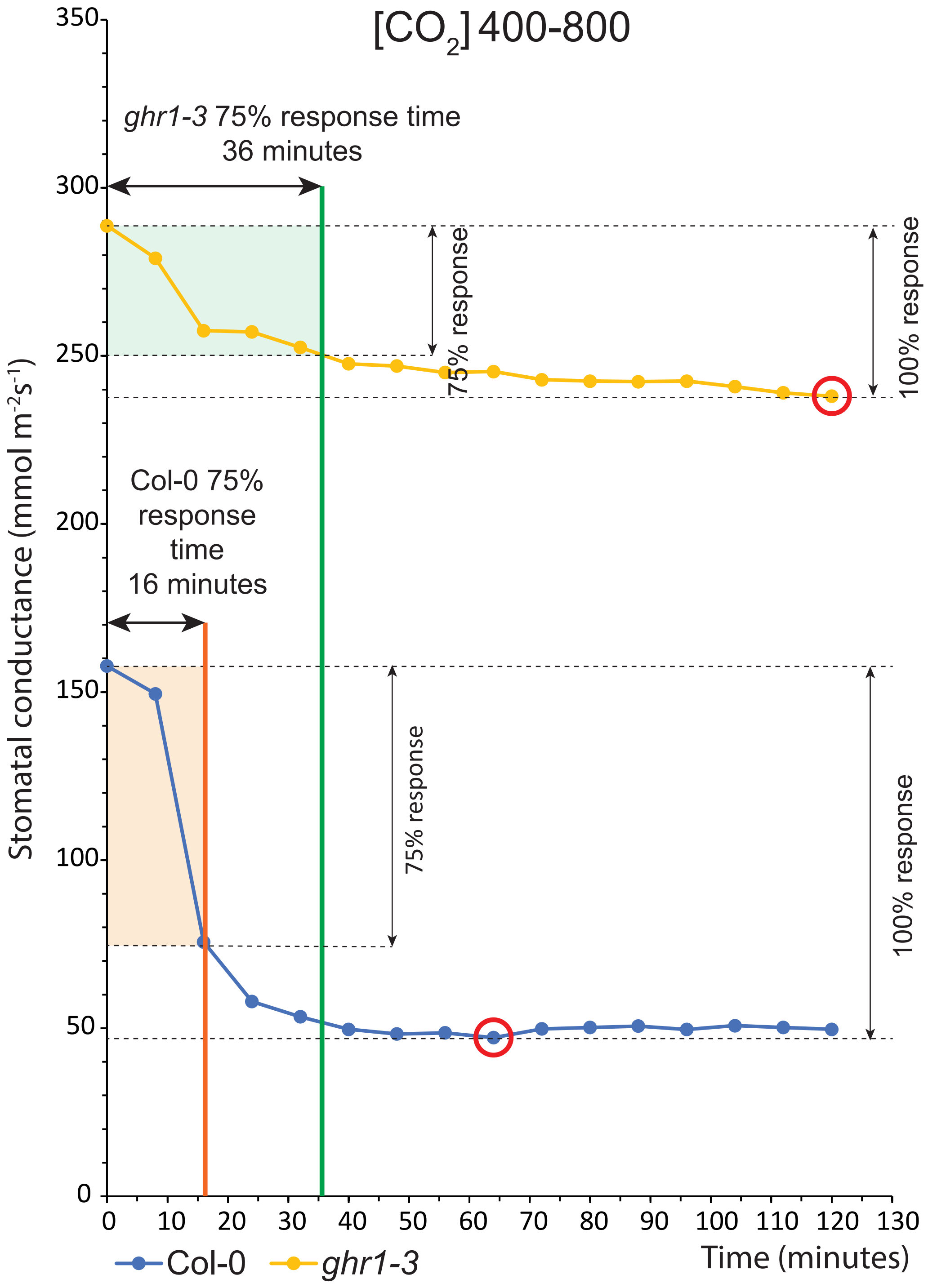

Supplementary Figure S1

Scheme explaining the calculation process for 75% stomatal response time using Col-0 and *ghr1-3* ambient to above ambient CO_2 stomatal responses as an example. Point chosen as the 100% stomatal response was always the one that had greatest change compared to pre-treatment value and is indicated with red circle on the schematic. Stomatal 75% response time is the amount of time it took for the plant stomata to reach 75% of the total response.

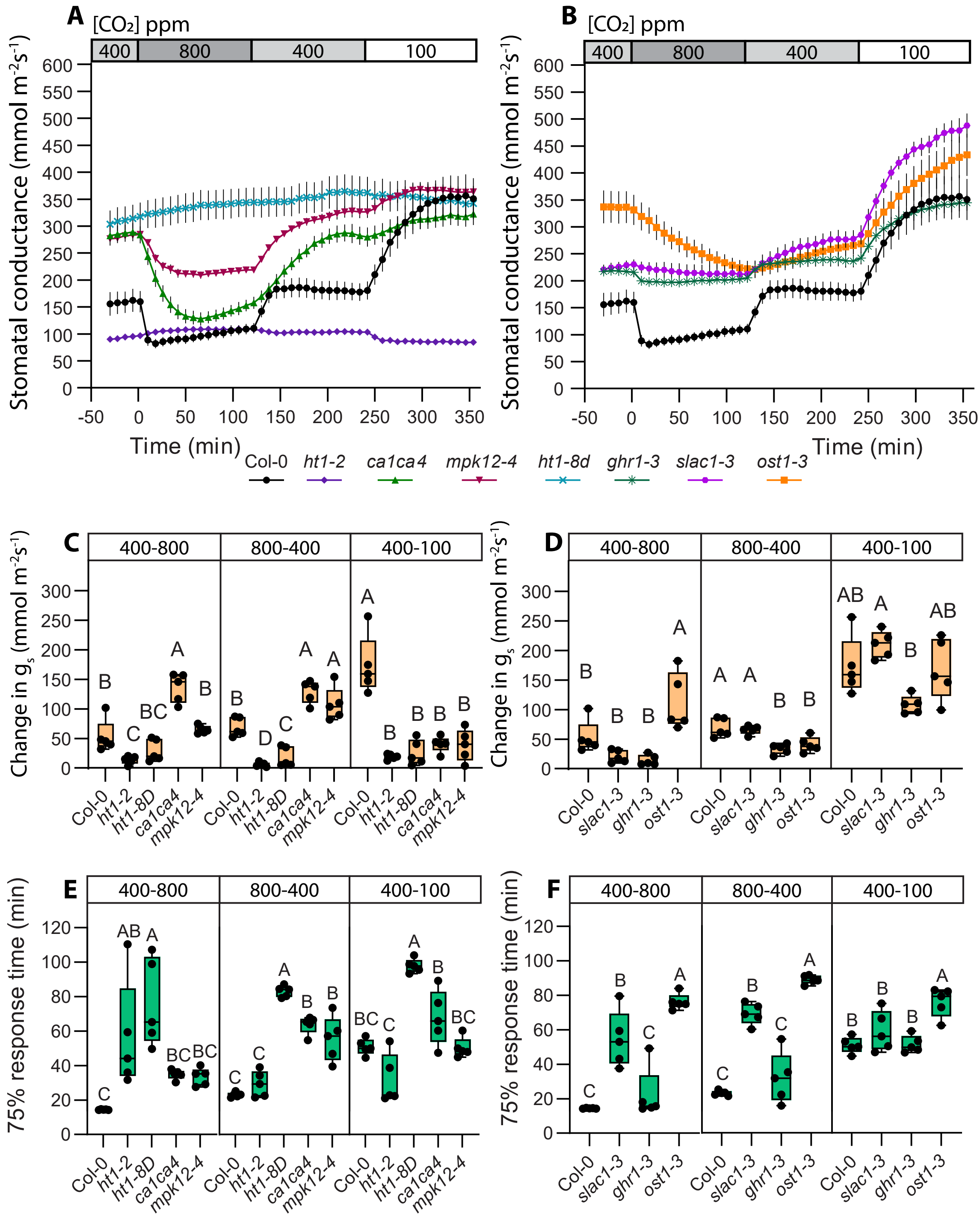

Supplementary Figure S2

Plant stomatal responses to 400-800-400-100 parts per million (ppm) CO_2 concentration changes. **(A)** and **(B)** Stomatal response to CO_2 concentration changes from 400 to 800 ppm, 800 to 400 ppm and 400 to 100 ppm, mean stomatal conductance ± SEM is shown. **(C,D)** Boxplot of stomatal conductance (g_s) change (mmol m^{-2} s^{-1}) in response to CO_2 concentration changes from 400 to 800 ppm, 800 to 400 ppm and 400 to 100 ppm, respectively. **(E,F)** Boxplot of 75% response time (minutes) of stomatal response to CO_2 concentration changes from 400 to 800 ppm, 800 to 400 ppm and 400 to 100 ppm, respectively. **(C-F)** Boxes represent 25-75 % quartiles and median as the horizontal lines, whiskers indicate the smallest and largest values, points show individual plant values. Statistically significantly different groups are marked with different letters (One-way ANOVA with Tukey *post hoc* test, $p < 0.05$). **(A-F)** Sample size was 5 for all plant lines. VPD during experiments was 0.9 kPa in **(A-F)**. Start of first treatment was between 11:30 to 12:30. Col-0 data is same as used in Figure 1, experiments with Col-0 and mutant lines were done together.
