# Supplementary material for: Stomatal CO2 responses at sub- and above-ambient CO2 levels employ different pathways in Arabidopsis
Source: Plant Physiol. 2024 Jun 4;196(1):608–20. doi: 10.1093/plphys/kiae320 (PMC11376393; doi:10.1093/plphys/kiae320)
Supplement: kiae320_Supplementary_Data [file pp.01350.2023-s03.pdf › pp.01350.2023-s03.pdf]

Plant Physiology®

Stomatal CO2-responses at sub- and above-ambient CO2 levels employ different pathways in Arabidopsis

Kaspar Koolmeister, Ebe Merilo, Hanna Hõrak, and Hannes Kollist

Review Timeline:

Submission Date:	29-Aug-2023
Editorial Decision:	05-Jan-2024
Revision Received:	08-Mar-2024
Editorial Decision:	03-Apr-2024
Revision Received:	03-May-2024
Accepted:	12-May-2024

05-Jan-2024

Dr. Hanna Hõrak
Tartu Ulikool
Institute of Technology
Tartu 50411
Estonia

RE: Stomatal CO2-responses at sub- and above-ambient CO2 levels employ different pathways in Arabidopsis

Dear Dr. Hõrak:

We have received reviews of your manuscript, which you may view at the end of this letter. On the basis of the advice received and my own evaluation, I believe that, pending major revision, this article may ultimately be acceptable for publication in Plant Physiology.

The reviewers found the topic of your MS interesting and appreciated the novelty and value of the data presented. However, both reviewers expressed concerns regarding the justification of the conclusions, and that this was due to both overinterpretation of data and language used to describe the findings. Particularly comments were raised regarding the model fits of the kinetics responses, with alternative assessment suggested that could lead to different conclusions. There were also several sections that need clarification. Given these considerations, the manuscript would benefit significantly from a major revision. This entails some reworking to address the concerns raised by the reviewers. Please ensure that all comments are addressed in a revised version.

When you are ready to submit your revision, click the below link and find the red arrow under your Author Tasks heading for this revision submission.

If you want to visit this version of the manuscript, you may click the link below and then click on the folder that reads "Post Decision Manuscripts".

Link Not Available

When uploading the revised version of this article, please be sure to include as complete an answer as possible to the referees' criticisms during the first round of reviews in the "response to reviewers" section.

Plant Physiology considers a revision for up to 60 days after a manuscript has been provisionally accepted; limited extension of this timeframe is at the discretion of the Monitoring Editor. A reminder notice will be sent to you 2 weeks before the deadline, at which time you may choose to contact the editorial office to request an extension; if so, please provide a justification for your request. After the expiration of this deadline, the article would need to be submitted as a resubmission and would be handled as such.

Thank you very much for giving us an opportunity to review this work. I look forward to receiving the next version.

Sincerely,

Editor
Plant Physiology

Want to add this revision deadline to your calendar? Click below!

--- Reviewer comments:
Reviewer #1 (Comments for the Author):

This manuscript compared stomatal responses to changes in CO2 concentration between wild-type and mutant arabidopsis, for a range of different ranges, directions and magnitudes of CO2 changes, and at two different VPDs. The authors found differences in the rate and amplitude of changes in stomatal conductance between shifts at sub-ambient (100-400 ppm) and above-ambient (400-800) ranges of CO2, and they also found that these differences were idiosyncratic across various mutants involved in CO2 signalling. Based on these results, the authors conclude that stomatal closure in response to CO2 "comprises two different underlying processes" for sub- and above-ambient CO2 concentration ranges.

I think this conclusion, as currently worded, is not justified, though it could be remedied by minor rewording. The authors' results in fact seem to show that all the same pathways are involved in the response in both CO2 ranges, albeit with differing degrees of importance. My main problem is that the authors are drawing binary conclusions (e.g., pathway or locus X is or is not involved in response Y) from continuous data. This is common in the guard cell biology literature, but it's wrong.

Here are two other specific examples of this fallacy in the current paper: Lines 206-7 conclude that HT1 is "crucial to initiate stomatal closure in response to CO2", yet HT1 mutants clearly have a CO2 response, albeit a weaker one, proving that HT1 is not essential for the response (though it clearly contributes). A similar statement is made on 255-6, where the authors conclude that "the [SLAC1] CO2-specific signaling pathway is essential for stomatal opening in the sub-ambient CO2 levels," based on reduced amplitude and rate of response in mutants. Yet what their results actually show is that some part of the response, and/or the overall magnitude and speed of the response, differ in the mutant; they do not show that the pathway is essential for the response. In fact, that the response occurs at all in the mutants proves that the underlying loci are *not* essential for the response.

These issues can be addressed by tempering the language used around these conclusions.

Another issue is how the authors quantified stomatal response kinetics, namely by fitting exponential functions to changes in stomatal conductance. This is fine in principle and many other studies have done the same or similar. However, the authors arbitrarily define some responses as "linear" and not exponential, and in consequence they do not report any response kinetics data for those responses. This distinction is quite subjective and, in my view, not supportable. I recommend that the authors instead use a model-independent measure of response kinetics (such as the time for 75% of the response to occur), and report results for every single response.

Finally, there are other possible (even likely) causal interpretations of differences in stomatal response kinetics between different CO2 ranges. For example, a given change of guard cell osmotic content will translate into very different changes in stomatal conductance when guard cell volume and turgor pressure are in different ranges (as one would expect in different CO2 concentration ranges), and this should lead to specific a priori predictions about the speed and magnitude of given responses. The authors omit any reference to this important context. They also omit any discussion of the role of processes outside of guard cells in the CO2 response, including extensive evidence that the responses are partly influenced by mesophyll processes (see Lawson and Matthews 2020, Ann Rev Plant Biol 71:273 for review).

In summary, I believe the experiments reported here are valuable and can provide insights useful to the stomatal research community. The manuscript is very well-written. However, the manuscript as currently written overstates some of the results, omits some relevant context, and contains some questionable analytical methods.

70-72: In fact, there is direct experimental evidence that high VPD can accelerate stomatal movements (e.g., Mott et al 1999, J Exp Bot 50:1207). Same issue again in the paragraph at lines 372-400.

134-136: A bit more detail is needed here; please give the specific exponential function used, and show (in the supplemental would be fine) some fitted vs measured results.

141-142: I'm not sure this is the best comparison, given that the shift between 400 and 800 ppm is one-third larger than that between 100 and 400 ppm.

Fig 2A, E: I don't understand why the k value for ca1ca4 is shown as "NA" for the 100-400 ppm transition, when from panel A, stomatal conductance declined substantially in that case (by about 30%). Same thing again for Fig 4A vs E: ca1ca4, mpk12-4 and ht1-8d all respond to the 400-100 transition identically to one another and nearly identically to Col-0, yet no values are shown for the corresponding k-values. Again for ost1-3 in Fig 5B/F. I suspect the authors didn't fit exponentials in these cases because they defined the responses as "linear"; indeed the responses aren't exactly exponential, but then neither are the responses of Col0. This seems like a very arbitrary and subjective distinction. I think the authors must either fit exponentials to every response, or, preferably, use a different, model-independent measure to quantify kinetics (e.g., the time for 75% of the overall response to occur).

203-204: I just don't see this in the data. The HT mutants clearly have a much weaker response, but they do respond to the 400-800 transition. This is clear at both VPDs. Thus the conclusion on 206-7 that HT1 is "crucial to initiate stomatal closure in response to CO2" seems unjustified. A similar statement is made on 255-6, based on reduced amplitude and rate of response in

mutants; those show that some part of the response, and/or the overall magnitude and speed of the response, differ in the mutant, but not that the pathway is essential for the response. In fact, the fact that the response occurs at all in the mutants proves that the underlying loci are *not* essential for the response.

228-229: "...linear functions are inherently slower than exponential." This statement is not meaningful. The 'speed' of an exponential response is ambiguous because it varies continuously during the response, so it can't be unambiguously compared with the speed of a linear response. More generally, a linear response can just as easily create a given finite change in a given finite period of time as an exponential response.

373: This is an odd reference for VPD effects; literally thousands of other papers, before and after Morison and Gifford (1983), reported the same response.

401: This is inaccurate as written. Elevated temperatures do not by themselves affect atmospheric dryness at all; they tend to increase leaf temperature, which increases leaf-to-air VPD, but they have no direct effect on atmospheric dryness (vapor pressure). You could say they increase the 'evaporative potential' or 'evaporative demand' of the atmosphere.

Reviewer #2 (Comments for the Author):

I have reviewed the manuscript by Koolmeister et al. entitled 'Stomatal CO2-responses at sub- and above-ambient CO2 levels employ different pathways in Arabidopsis'. The question here is to distinguish the pathways governing stomatal movements at sub-ambient and above-ambient CO2, by phenotyping known Arabidopsis mutants for their stomatal dynamics in response to various CO2 sequences. The manuscript presents original data on 7 mutants thoroughly assessed through a common protocol designed to explore stomatal response to CO2 in the low and high range, testing different sequence orders (400-100-400-800, 400-800-400-100, 400-800-100 and 400-100-800). Moreover, a number of dynamics have also been monitored at high VPD (obtained here through low RH). The data are valuable for the community. However, their analysis is flawed due to inappropriate use of exponential fit (see major point 1 below), making some interpretation unreliable. I strongly recommend to present the initial slope of change in gs (0-10 min) instead of the k-value.

Some results will then look differently: the 100-400 closure will not appear much slower than the 400-800 stomatal closure; stomata will not present faster CO2-responsiveness at high VPD; SLAC1 and GHR1 will probably not be considered as more important for above-ambient CO2-induced stomatal closure. Other conclusions should remain unaffected: no major effects of the order of CO2 sequence (to my knowledge, this has never been tested so carefully); crucial role of HT1 irrespective of CO2 levels (confirmatory); lower importance of MPK12 and carbonic anhydrases for stomatal closure at high CO2.

Major points

1) The use of the k parameter obtained from exponential fit is misleading. First, the exponential form implies that the rate of change of gs is proportional to the difference between the actual and the final state. But at start, when the initial state is far from the final state, stomata certainly cannot achieve the high rate that would be dictated by an exponential response, because stomata are subjected to biophysical limits (i.e. set by solute transporters, water channels...). For instance, let us look at Fig. 1A/B 400-100: in the first 15 min following CO2 change, the rates of change in gs are similar between high and low VPD, probably reflecting the maximal speed of stomatal opening achievable in these conditions. But because the amplitude is lower at high VPD, its apparent k looks higher. In this system, there is covariation between the amplitude of response and the k parameter. Looking at the initial slope (dgs / dt) would be more relevant here. Another example is Fig. 2A/C/E, Col-0 vs. mpk12-4: quite incredibly, the authors write that "the response of mpk12-4 to the 100-400 transition was as fast as in wild-type, but significantly smaller in magnitude (Fig. 2A,C,E)" (lines 202-203). How can it be stated that the response of mpk12 is as fast as in Col-0, when the raw data show that after 15 min gs has already dropped by 150 mmol m-2 s-1 in the wild-type but has not started to vary in the mutant?! Clearly, the exponential fit returns a k parameter that does not reflect the actual speed, because the biological processes are not purely exponential and generate covariation between k and amplitude upon exponential fitting.

Second, the k parameter is derived from kinetics that sometimes do not look exponential (e.g. Fig. 1A 100-400, Fig. 1C 400-800...). Showing the fitted curves here would certainly not be convincing. Again, using the initial slope would be better. Curves considered as linear in the current version could equally be analysed in this way.

Third, the temporal resolution (8 min as can be inferred from the graphs) is not sufficient to properly estimate rapid responses with high k values (up to 0.1 min-1, meaning that 63% of the response is reached within 10 min). To say it differently, one cannot reasonably fit an exponential curve when the plateau is reached at the first point (e.g. Fig 1E 400-800). In such cases, the initial slope will also probably underestimate the actual responsiveness (so it would be okay to use this estimate provided the risk of underestimation is stated in the text).

2) Taking the previous point into account, I do not see any strong interaction between VPD and CO_2 response: all lines respond with lower magnitude, but similar dynamics. I recommend removing from the text all statements of interaction between VPD and CO_2 response. For instance, lines 248-250: "A remarkable exception was ghr1-3: stomatal response to 400-800 was completely missing under elevated VPD in ghr1-3". Actually the breaking point at 400-800 for ghr1-3 is difficult to see at control VPD, and is still harder to detect at high VPD because the amplitude is reduced like in the other lines. So it does not look as an exception. This result remains interesting, yet. On the other hand, the discussion could be improved by referring to previous articles (beyond Morison and Gifford 1983) like Bunce 1998 PCE or Talbott et al 2003 JXB. There is also a number of papers on the interaction between ABA and CO_2 (discussed in the penultimate paragraph) following Raschke 1975 Planta.

3) I appreciate the value of showing the response of many previously known mutants in a single, new study. However, it is not always clear which phenotypes are new and which ones are confirmatory. For instance, on mpk12, some CO_2 sequences reported here were already tested in Jakobson et al 2016 PLoS Biol and in Takahashi et al 2022 Sci Adv. The latter phenotype is mentioned quite late in the text (line 319), but the former is not. For ca1 ca4, some comparable CO_2 responses have been published by Hu et al 2010 Nat Cell Biol and Hu et al 2015 Plant Physiol, etc... Please consider adding a table reviewing the CO_2 sequences already published for the different mutants, and discuss the consistency with your own results. This would better highlight the novelty of your paper.

Minor points

- The title of the manuscript ('Stomatal CO_2-responses at sub- and above-ambient CO_2 levels employ different pathways in Arabidopsis') does not read well. I would suggest something like 'Distinct pathways for stomatal responses to high or low CO_2'.
- Fig 1: align panel letters horizontally
- Lines 166-167: "and essentially masks the latter in the 800-100 transition" unclear, consider deleting or reformulating.
- Lines 228-229: "linear functions are inherently slower than exponential" this is not true, please remove (linear functions with high slope may be faster than exponential functions with low k)
- Lines 253-263: the 400-100 stomatal opening period should have been commented before (I was confused not to find it in the first description of Fig. 2 and Fig. 3).
- Lines 264-297: this section is really hard to follow, please consider rewriting it

Dear Dr. ,

We are glad to resubmit our revised manuscript 'Stomatal CO_2-responses at sub- and above-ambient CO_2 levels employ different pathways in Arabidopsis' for consideration for publication in *Plant Physiology*. We are grateful to the reviewers for their thoughtful comments and suggestions. We have thoroughly revised the manuscript according to the reviewers' suggestions and provide point-by-point answers to their comments below in blue colour. The changes in the manuscript are highlighted in yellow background**, line numbers are according to the highlighted manuscript file.**

Thank you for your time and consideration,

Hanna Hõrak

-- Reviewer comments:

Reviewer #1 (Comments for the Author):

This manuscript compared stomatal responses to changes in CO2 concentration between wild-type and mutant arabidopsis, for a range of different ranges, directions and magnitudes of CO2 changes, and at two different VPDs. The authors found differences in the rate and amplitude of changes in stomatal conductance between shifts at sub-ambient (100-400 ppm) and above-ambient (400-800) ranges of CO2, and they also found that these differences were idiosyncratic across various mutants involved in CO2 signalling. Based on these results, the authors conclude that stomatal closure in response to CO2 "comprises two different underlying processes" for sub- and above-ambient CO2 concentration ranges.

I think this conclusion, as currently worded, is not justified, though it could be remedied by minor rewording. The authors' results in fact seem to show that all the same pathways are involved in the response in both CO2 ranges, albeit with differing degrees of importance. My main problem is that the authors are drawing binary conclusions (e.g., pathway or locus X is or is not involved in response Y) from continuous data. This is common in the guard cell biology literature, but it's wrong.

We agree with the reviewer that both studied pathways are involved in stomatal CO_2 responses under both studied concentration ranges but with different degrees of importance. We have reworded the conclusion in lines 309-312 and addressed the problem of drawing strong binary conclusions by rewordings throughout the manuscript, where appropriate.

Here are two other specific examples of this fallacy in the current paper: Lines 206-7 conclude that HT1 is "crucial to initiate stomatal closure in response to CO2", yet HT1 mutants clearly have a CO2 response, albeit a weaker one, proving that HT1 is not essential for the response (though it clearly contributes). A similar statement is made on 255-6, where the authors conclude that "the [SLAC1] CO2-specific signaling pathway is essential for stomatal opening in the sub-ambient CO2 levels," based on reduced amplitude and rate of response in mutants. Yet what their results actually show is that some part of the response, and/or the overall magnitude and speed of the response, differ in the mutant; they do not show that the pathway is essential for the response. In fact, that the response occurs at all in the mutants proves that the underlying loci are *not* essential for the response.

We have revised the indicated lines to refrain from too categorical claims, currently in lines 200-201 and 221-223.

These issues can be addressed by tempering the language used around these conclusions.

Another issue is how the authors quantified stomatal response kinetics, namely by fitting exponential functions to changes in stomatal conductance. This is fine in principle and many other studies have done the same or similar. However, the authors arbitrarily define some responses as "linear" and not exponential, and in consequence they do not report any response kinetics data for those responses. This distinction is quite subjective and, in my view, not supportable. I recommend that the authors instead use a model-independent measure of response kinetics (such as the time for 75% of the response to occur), and report results for every single response.

We thank the reviewer for the suggestion. We have now replaced the previously shown k-values with the 75% response times as a model-independent measure of response kinetics, as suggested. While this parameter can technically be calculated for all responses, it is not informative for lines with very small, if any stomatal responses (e.g *ht1-2* and *ht1-8D* in the 100-400 transition), as we have also indicated in the manuscript (lines 196-198). In other cases, we believe the 75% response times to be informative for meaningful comparisons of stomatal response speeds.

Finally, there are other possible (even likely) causal interpretations of differences in stomatal response kinetics between different CO2 ranges. For example, a given change of guard cell osmotic content will translate into very different changes in stomatal conductance when guard cell volume and turgor pressure are in different ranges (as one would expect in different CO2 concentration ranges), and this should lead to specific a priori predictions about the speed and magnitude of given responses. The authors omit any reference to this important context. They also omit any discussion of the role of processes outside of guard cells in the CO2 response, including extensive evidence that the responses are partly influenced by mesophyll processes (see

and Matthews 2020, Ann Rev Plant Biol 71:273 for review).

We agree with the reviewer that guard cell aperture changes at different ranges of turgor pressure are very important but equally very hard to account for in experiments. We have included a sentence on guard cell volume and stomatal apertures in lines 314-317 and added discussion on mesophyll influence on stomatal CO_2 responses in lines 423-434.

In summary, I believe the experiments reported here are valuable and can provide insights useful to the stomatal research community. The manuscript is very well-written. However, the manuscript as currently written overstates some of the results, omits some relevant context, and contains some questionable analytical methods.

We thank the reviewer for pointing out the value of our data. We now took extra care to avoid potential overstatements throughout the manuscript.

70-72: In fact, there is direct experimental evidence that high VPD can accelerate stomatal movements (e.g., Mott et al 1999, J Exp Bot 50:1207). Same issue again in the paragraph at lines 372-400.

We thank the reviewer for drawing our attention to this reference. We have discussed the reference and another recent one on a similar topic now in lines 64-67 and 393-397.

134-136: A bit more detail is needed here; please give the specific exponential function used, and show (in the supplemental would be fine) some fitted vs measured results.

We have now replaced the exponential fits with the calculation of 75% response times as specified in the methods section in lines 129-132.

141-142: I'm not sure this is the best comparison, given that the shift between 400 and 800 ppm is one-third larger than that between 100 and 400 ppm.

We have reworded these lines (now 137-138) to stress the difference between sub-ambient to ambient and ambient to above-ambient CO_2 concentration transitions that were the focus of our approach.

Fig 2A, E: I don't understand why the k value for ca1ca4 is shown as "NA" for the 100-400 ppm transition, when from panel A, stomatal conductance declined substantially in that case (by about 30%). Same thing again for Fig 4A vs E: ca1ca4, mpk12-4 and ht1-8d all respond to the 400-100 transition identically to one another and nearly identically to Col-0, yet no values are shown for the corresponding k-values. Again for ost1-3 in Fig 5B/F. I suspect the authors didn't fit exponentials in these cases because they defined the responses as "linear"; indeed the responses aren't exactly exponential, but then neither are the responses of Col0. This seems like a very arbitrary and subjective distinction. I think the authors must either fit exponentials to every response, or, preferably, use a different, model-independent measure to quantify kinetics (e.g., the time for 75% of the overall response to occur).

We have now instead used a model-independent measure to quantify response speed (the time for 75% of the overall response to occur) as suggested by the reviewer.

203-204: I just don't see this in the data. The HT mutants clearly have a much weaker response, but they do respond to the 400-800 transition. This is clear at both VPDs. Thus the conclusion on 206-7 that HT1 is "crucial to initiate stomatal closure in response to CO2" seems unjustified. A similar statement is made on 255-6, based on reduced amplitude and rate of response in mutants; those show that some part of the response, and/or the overall magnitude and speed of the response, differ in the mutant, but not that the pathway is essential for the response. In fact, the fact that the response occurs at all in the mutants proves that the underlying loci are *not* essential for the response.

We have revised the text to remove the described categorical claims, currently in lines 200-201, 203-204, and 221-223.

228-229: "...linear functions are inherently slower than exponential." This statement is not meaningful. The 'speed' of an exponential response is ambiguous because it varies continuously during the response, so it can't be unambiguously compared with the speed of a linear response. More generally, a linear response can just as easily create a given finite change in a given finite period of time as an exponential response.

We have removed these lines, as we have replaced curve fits with 75% response time as suggested by the reviewer.

373: This is an odd reference for VPD effects; literally thousands of other papers, before and after Morison and Gifford (1983), reported the same response.

We have added further relevant references on the topic of VPD and CO_2-responsiveness in lines 387-391.

401: This is inaccurate as written. Elevated temperatures do not by themselves affect atmospheric dryness at all; they tend to increase leaf temperature, which increases leaf-to-air VPD, but they have no direct effect on atmospheric dryness (vapor pressure). You could say they increase the 'evaporative potential' or 'evaporative demand' of the atmosphere.

We have revised the line as suggested (now lines 435-436).

Reviewer #2 (Comments for the Author):

I have reviewed the manuscript by Koolmeister et al. entitled 'Stomatal CO2-responses at sub- and above-ambient CO2 levels employ different pathways in Arabidopsis'. The question here is to distinguish the pathways governing stomatal movements at sub-ambient and above-ambient CO2, by phenotyping known Arabidopsis mutants for their stomatal dynamics in response to various CO2 sequences. The manuscript presents original data on 7 mutants thoroughly assessed through a common protocol designed to explore stomatal response to CO2 in the low and high range, testing different sequence orders (400-100-400-800, 400-800-400-100, 400-800-100 and 400-100-800). Moreover, a number of dynamics have also been monitored at high VPD (obtained here through low RH). The data are valuable for the community. However, their analysis is flawed due to inappropriate use of exponential fit (see major point 1 below), making some interpretation unreliable. I strongly recommend to present the initial slope of change in gs (0-10 min) instead of the k-value.

We thank the reviewer for the comments. We have now replaced the k-values with a model-independent measure to quantify kinetics, the time for 75% of the overall response to occur, which is applicable to all mutants. We appreciate the suggestion to use the initial slope of the reaction and agree that it would be a good measure to describe initial stomatal response kinetics. However, the 75% response time takes into account response speed across a longer timeframe and we consider this important to describe the speed of the overall stomatal response that occurs throughout two hours.

Some results will then look differently: the 100-400 closure will not appear much slower than the 400-800 stomatal closure; stomata will not present faster CO2-responsiveness at high VPD; SLAC1 and GHR1 will probably not be considered as more important for above-ambient CO2-induced stomatal closure. Other conclusions should remain unaffected: no major effects of the order of CO2 sequence (to my knowledge, this has never been tested so carefully); crucial role of HT1 irrespective of CO2 levels (confirmatory); lower importance of MPK12 and carbonic anhydrases for stomatal closure at high CO2.

We have now replaced the k-values with the 75% response times. Most of our conclusions remain the same as based on the model fits and k-values, except that elevated VPD did not accelerate CO_2-induced stomatal closure in the 400-800 transition, although 100-400, 400-100, and 800-100 responses were faster under elevated VPD (Figure 1B,F).

Major points

1) The use of the k parameter obtained from exponential fit is misleading. First, the exponential form implies that the rate of change of gs is proportional to the difference between the actual and the final state. But at start, when the initial state is far from the final state, stomata certainly cannot achieve the high rate that would be dictated by an exponential response, because stomata are subjected to biophysical limits (i.e. set by solute transporters, water channels...). For instance, let us look at Fig. 1A/B 400-100: in the first 15 min following CO2 change, the rates of change in gs are similar between high and low VPD, probably reflecting the maximal speed of stomatal opening achievable in these conditions. But because the amplitude is lower at high VPD, its apparent k looks higher. In this system, there is covariation between the amplitude of response and the k parameter. Looking at the initial slope (dgs / dt) would be more relevant here. Another example is Fig. 2A/C/E, Col-0 vs. mpk12-4: quite incredibly, the authors write that "the response of mpk12-4 to the 100-400 transition was as fast as in wild-type, but significantly smaller in magnitude (Fig. 2A,C,E)" (lines 202-203). How can it be stated that the response of mpk12 is as fast as in Col-0, when the raw data show that after 15 min gs has already dropped by 150 mmol m-2 s-1 in the wild-type but has not started to vary in the mutant?! Clearly, the exponential fit returns a k parameter that does not reflect the actual speed, because the biological processes are not purely exponential and generate covariation between k and amplitude upon exponential fitting.

We agree with the reviewer that the k-value that was used in the first version of the manuscript was not the best parameter as it was not applicable in case of all responses. We tried different model-independent approaches, including initial slope calculation (dgs/dt) and reached the conclusion that the time for 75% of the overall response to occur is most informative for our experimental set-ups. This parameter does not describe the fast kinetics within first minutes, but here we wanted to compare not the initial fast response rates, but the speeds of the overall two-hour responses between genotypes and therefore used the 75% response time.

Second, the k parameter is derived from kinetics that sometimes do not look exponential (e.g. Fig. 1A 100-400, Fig. 1C 400-800...). Showing the fitted curves here would certainly not be convincing. Again, using the initial slope would be better. Curves considered as linear in the current version could equally be analysed in this way.

We have now replaced the k-values with another model-independent measure to quantify response speeds (the time for 75% of the overall response to occur).

Third, the temporal resolution (8 min as can be inferred from the graphs) is not sufficient to properly estimate rapid responses with high k values (up to 0.1 min-1, meaning that 63% of the response is reached within 10 min). To say it differently, one cannot reasonably fit an exponential curve when the plateau is reached at the first point (e.g. Fig 1E 400-800). In such cases, the initial slope will also probably underestimate the actual responsiveness (so it would be okay to use this estimate provided the risk of underestimation is stated in the text).

We agree with the reviewer that the 8-minute resolution might not be the best to quantify initial response kinetics, hence we have now used 75% response times to describe overall response speed across the two-hour response window.

2) Taking the previous point into account, I do not see any strong interaction between VPD and CO2 response: all lines respond with lower magnitude, but similar dynamics. I recommend removing from

the text all statements of interaction between VPD and CO2 response. For instance, lines 248-250: "A remarkable exception was ghr1-3: stomatal response to 400-800 was completely missing under elevated VPD in ghr1-3". Actually the breaking point at 400-800 for ghr1-3 is difficult to see at control VPD, and is still harder to detect at high VPD because the amplitude is reduced like in the other lines. So it does not look as an exception. This result remains interesting, yet. On the other hand, the discussion could be improved by referring to previous articles (beyond Morison and Gifford 1983) like Bunce 1998 PCE or Talbott et al 2003 JXB. There is also a number of papers on the interaction between ABA and CO2 (discussed in the penultimate paragraph) following Raschke 1975 Planta.

We respectfully disagree with the reviewer on the presence of interaction between VPD and CO_2 responses. The 75% response times are shorter under elevated VPD in the 100-400, 400-100, and 800-100 transitions in wild-type plants (Figure 1B,F). We also see differences compared with wild-type in some mutants' 75% response speeds under elevated VPD, indicating that there is some interaction between the CO_2 and VPD responses (Figure 2E,F, 3E,F, lines 211-223, 253-255, and 258-264). We also believe that the near lack of stomatal closure under the 400-800 transition in the *ghr1-3* is biologically meaningful, as discussed in lines 416-422. We thank the reviewer for pointing out relevant references that also support an interaction between VPD and CO_2 responses, we have now included these in the discussion in lines 385-391.

3) I appreciate the value of showing the response of many previously known mutants in a single, new study. However, it is not always clear which phenotypes are new and which ones are confirmatory. For instance, on mpk12, some CO2 sequences reported here were already tested in Jakobson et al 2016 PLoS Biol and in Takahashi et al 2022 Sci Adv. The latter phenotype is mentioned quite late in the text (line 319), but the former is not. For ca1 ca4, some comparable CO2 responses have been published by Hu et al 2010 Nat Cell Biol and Hu et al 2015 Plant Physiol, etc... Please consider adding a table reviewing the CO2 sequences already published for the different mutants, and discuss the consistency with your own results. This would better highlight the novelty of your paper.

We have added a table (Table 1) combining previously published results for the CO_2 responses of our studied mutants and our results, highlighting our added contribution and consistency of previous results with our results, now discussed in lines 295-306.

Minor points

- The title of the manuscript ('Stomatal CO2-responses at sub- and above-ambient CO2 levels employ different pathways in Arabidopsis') does not read well. I would suggest something like 'Distinct pathways for stomatal responses to high or low CO2'.

We have kept the title as is, as we consider the distinction of sub- and above-ambient CO_2 concentration ranges important for studying stomatal CO_2 responses, and one of our messages is that "high or low CO_2" alone is not meaningful, but it matters, in which CO_2 concentration ranges the elevation or reduction of CO_2 levels is carried out.

- Fig 1: align panel letters horizontally

We have revised the figure and aligned letters.

- Lines 166-167: "and essentially masks the latter in the 800-100 transition" unclear, consider deleting or reformulating.

We have deleted this phrase.

- Lines 228-229: "linear functions are inherently slower than exponential" this is not true, please remove (linear functions with high slope may be faster than exponential functions with low k)

We have removed the statement.

- Lines 253-263: the 400-100 stomatal opening period should have been commented before (I was confused not to find it in the first description of Fig. 2 and Fig. 3).

We have reorganised the text to comment on the stomatal opening responses in the same sections as the closure responses.

- Lines 264-297: this section is really hard to follow, please consider rewriting it

We have revised this section to make it easier to follow.

03-Apr-2024

Dr. Hanna Hõrak
Tartu Ulikool
Institute of Technology
Tartu 50411
Estonia

RE: Stomatal CO2-responses at sub- and above-ambient CO2 levels employ different pathways in Arabidopsis

Dear Dr. Hanna Hõrak:

We are pleased to accept your manuscript for publication in the Plant Physiology. This acceptance is contingent on revision based on the comments of our reviewers. In particular, please consider the following:

responses and has queried the interpretation of using the 75% response time as speed alone, as it also includes the magnitude of response. To address these concerns, I think that it would be beneficial to include a clear explanation of this method and how it relates to speed and magnitude along with any potential caveat. It may be beneficial to provide a graphical illustration in the supplemental section to illustrate this. It is also important that language is nuanced in a way to guide the reader ensuring clear and accurate interpretation of the results, and that any implications for the final conclusions are transparent. Reviewer 2 has additionally provided several minor suggestions that will enhance the manuscript. I look forward to receiving your final MS.

Please highlight all changes and include a detailed annotation to changes to the text, with line numbers, and noting your responses to the comments.

To submit your revised manuscript, click:

Link Not Available

If you cannot return the revised manuscript within 8 weeks of receipt, please let us know. Otherwise, we will assume that you have elected not to revise the manuscript and are withdrawing it.

Thank you for allowing us to review your work. We look forward to hearing from you soon.

Sincerely,

Editor
Plant Physiology

-- Reviewer comments:
Reviewer #1 (Comments for the Author):

Thank you for addressing my concerns from the earlier draft. The revised version is much improved, and I look forward to sharing it with my students and colleagues.

Reviewer #2 (Comments for the Author):

I have reviewed the revised manuscript. This version has been improved and I thank the authors for having accounted for most of the comments that were raised in the first version. It is clear from the new Table 1 to what extent the data presented here are novel compared to what is available in the literature.

That being said, I still disagree with some conclusions because of the new metrics used to quantify stomatal responsiveness - the time to reach 75% of the response - is still misleading. This metrics integrates both speed and magnitude, while in the text it is mostly interpreted as a measure of speed. For instance, I cannot agree with the statement that "the 400-800 stomatal closure was faster than 100-400 closure under normal VPD" (lines 153-154): Fig 1A essentially show similar initial speed of closure for both conditions, but lower magnitude for 400-800. Similarly, the initial speed of opening for 400-100 looks similar as the one for 800-400 (Fig 1C), while it is reported to be slower (line 162). Same problem when assessing the effect of high VPD: the kinetics

essentially show that the CO_2 responses are conserved at high VPD, yet with lower magnitude. Fig 2C-F shows that the 75% response time largely mirrors the amplitude, illustrating that both parameters are not independent. Should the authors stick to this metrics, I strongly recommend not using terms related to speed (i.e. avoiding terms like "slow" or "fast"). Alternatively (better option), the authors may systematically use the initial slope (which is largely unaffected by the amplitude) and the 2-h amplitude.

Minor points

- I would still suggest to find a more legible title.

- lines 31-32: I don't like the concluding sentence "Hence, multi-factor treatments are needed to understand plant behavior under future climate conditions." which sounds a little bit like a commonplace (while it could be argued that abrupt changes in CO_2 concentration do not reflect future climate conditions...)

- The references that have been added in the text of the second version do not appear in the ref list (e.g. Mott et al 1999, Pichaco et al 2024...)

- lines 72-73: "elevated VPD might prime plant stomata for fast closure via increased ABA concentration" The effect described here is a direct effect, it is not about priming

- lines 129-132: it is not clear whether data were normalized according to the initial vs. final gs or according to the minimal vs. maximal gs of the interval. It needs to be defined because several kinetics are not monotonic.

- In the same vein, it is not clear why on Fig 1D all t_75% points equal 16 min for the 400-800 sequence, while on Fig 1C the minimal value for this sequence is observed at 16 min after CO_2 has changed.

- lines 174-175: "400-800 ppm [CO_2] transition 75% response time was not affected by high VPD (Fig. 1A,B,E,F)". The temporal resolution and the range (close to zero) makes it impossible to detect a potential acceleration of stomatal closure due to VPD. So it should be reformulated (e.g. "we were not able to detect..." rather than "was not affected...") and the conclusion that "VPD has a minor role in above-ambient CO_2-induced stomatal closure" (lines 177-178) be omitted.

- lines 221-223: "Thus, stomatal opening response to sub-ambient [CO_2] is affected by all of the CO_2 signaling pathway components represented in this study." Unclear sentence.

- lines 412-414: "These data indicate that elevated ABA levels alone are not sufficient to explain the decrease of stomatal conductance under elevated VPD conditions." It is worth mentioning the OnGuard model showing ABA is not required for VPD-induced stomatal closure (Wang et al 2017 Plant Cell 10.1105/tpc.17.00694).

- lines 437-438: "changes in atmospheric VPD and CO_2 levels are perhaps the greatest agricultural challenges of the future" What about drought stress?

Dear Dr ,

We are submitting our revised manuscript, where we have made the changes suggested by you and reviewer 2. Specifically, we clarified the data analysis section to better explain the calculation of 75% response times and also added a Supplementary Figure 1 to graphically illustrate this method. We also incorporated the minor suggestions from reviewer 2. Changes to the text are highlighted in yellow colour and responses to reviewer 2 have been provided below in blue text. **Line numbers are according to the highlighted version of the manuscript.**

Thank you for your time and consideration,

Hanna Hõrak

-- Reviewer comments:

Reviewer #1 (Comments for the Author):

Thank you for addressing my concerns from the earlier draft. The revised version is much improved, and I look forward to sharing it with my students and colleagues.

Reviewer #2 (Comments for the Author):

I have reviewed the revised manuscript. This version has been improved and I thank the authors for having accounted for most of the comments that were raised in the first version. It is clear from the new Table 1 to what extent the data presented here are novel compared to what is available in the literature.

That being said, I still disagree with some conclusions because of the new metrics used to quantify stomatal responsiveness - the time to reach 75% of the response - is still misleading. This metrics integrates both speed and magnitude, while in the text it is mostly interpreted as a measure of speed. For instance, I cannot agree with the statement that "the 400-800 stomatal closure was faster than 100-400 closure under normal VPD" (lines 153-154): Fig 1A essentially show similar initial speed of closure for both conditions, but lower magnitude for 400-800. Similarly, the initial speed of opening for 400-100 looks similar as the one for 800-400 (Fig 1C), while it is reported to be slower (line 162). Same problem when assessing the effect of high VPD: the kinetics essentially show that the CO2 responses are conserved at high VPD, yet with lower magnitude. Fig 2C-F shows that the 75% response time largely mirrors the amplitude, illustrating that both parameters are not independent. Should the authors stick to this metrics, I strongly recommend not using terms related to speed (i.e. avoiding terms like "slow" or "fast"). Alternatively (better option), the authors may systematically use the initial slope (which is largely unaffected by the amplitude) and the 2-h amplitude.

We have clarified the methods section to better explain how 75% response time was calculated (lines 132-136) and also illustrated the method with a figure (Supplementary Figure 1). To acknowledge the caveats of the method, we also included the sentence “The 75% response time describes the overall response speed across the 2-hour treatment time but not the initial kinetics of

the stomatal responses” (lines 136-138). We have now added a sentence in lines 157-159 to indicate how stomatal response speed is defined in this manuscript with a reference to its calculation in the methods section and Supplementary Figure 1. It is true that as the 75% response time calculation is based on the maximal amplitude of the stomatal response, it is not independent of stomatal response amplitude. Yet, the maximal response can occur at different time points, describing different kinetics of the response that is captured by the 75% response time. Comparisons of stomatal 75% response times are often different from comparisons of response amplitude as illustrated by e.g. Figure 3C-F. Thus, we think that showing the 75% response time in addition to the response amplitude has additional value for describing the overall stomatal response speed, despite not showing the initial kinetics of first minutes of the response.

Minor points

- I would still suggest to find a more legible title.

We thank the reviewer for the suggestion. We believe the title describes our main finding as shortly as possible and thus have kept the title.

- lines 31-32: I don't like the concluding sentence "Hence, multi-factor treatments are needed to understand plant behavior under future climate conditions." which sounds a little bit like a commonplace (while it could be argued that abrupt changes in CO2 concentration do not reflect future climate conditions...)

We have now revised the final sentence of the abstract to read „Hence, multi-factor treatments are needed to understand how plants integrate different environmental signals and translate them to stomatal responses.”

- The references that have been added in the text of the second version do not appear in the ref list (e.g. Mott et al 1999, Pichaco et al 2024...)

We thank the reviewer for pointing out this error, we have now included these and several other references that had gone missing from our reference list due to an unexpected referencing software error.

- lines 72-73: "elevated VPD might prime plant stomata for fast closure via increased ABA concentration" The effect described here is a direct effect, it is not about priming

We have rewritten the sentence (in lines 71-74) to indicate the role of ABA in VPD-induced stomatal closure. It now reads “VPD has a direct effect on ABA concentration as increased ABA levels in angiosperms were observed 20 minutes after increasing VPD from 0.7 to 1.5 kPa (McAdam and Brodribb, 2015), promoting the conclusion that ABA may be involved in elevated VPD-induced stomatal closure in angiosperms (McAdam et al., 2016).”

- lines 129-132: it is not clear whether data were normalized according to the initial vs. final gs or according to the minimal vs. maximal gs of the interval. It needs to be defined because several kinetics are not monotonic.

To calculate the 75% stomatal response time, we used the gs difference between the time point before treatment and the point when maximal stomatal response had occurred, which was not always the final time point. We have revised the text in the data analysis section (lines 132-136) to clarify this and also added an illustration as Supplementary Figure 1 to show how the 75% response time was calculated.

- In the same vein, it is not clear why on Fig 1D all t_75% points equal 16 min for the 400-800 sequence, while on Fig 1C the minimal value for this sequence is observed at 16 min after CO2 has changed.

To calculate the 75% stomatal response time, we used the gs difference between the time point before treatment and the point when maximal stomatal response had occurred – for the 400-800 transition in Figures 1C-D, this was at time point 24, and 75% response time was on average 14.42 min (range 14.21 - 14.68 min), calculated as now clarified in data analysis section (lines 132-136) and in Supplementary Figure 1.

- lines 174-175: "400-800 ppm [CO2] transition 75% response time was not affected by high VPD (Fig. 1A,B,E,F)". The temporal resolution and the range (close to zero) makes it impossible to detect a potential acceleration of stomatal closure due to VPD. So it should be reformulated (e.g. "we were not able to detect..." rather than "was not affected...") and the conclusion that "VPD has a minor role in above-ambient CO2-induced stomatal closure" (lines 177-178) be omitted.

We have revised the text as suggested by the reviewer (lines 182-186).

- lines 221-223: "Thus, stomatal opening response to sub-ambient [CO2] is affected by all of the CO2 signaling pathway components represented in this study." Unclear sentence.

We have rewritten the sentence now in lines 229-231 to read "Thus, stomatal opening response to sub-ambient [CO_2] involves all of the CO_2 signaling pathway components represented in this study, including the carbonic anhydrases CA1 and CA4, and the HT1 and MPK12 kinases).".

- lines 412-414: "These data indicate that elevated ABA levels alone are not sufficient to explain the decrease of stomatal conductance under elevated VPD conditions." It is worth mentioning the OnGuard model showing ABA is not required for VPD-induced stomatal closure (Wang et al 2017 Plant Cell 10.1105/tpc.17.00694).

We have added the suggested reference, the sentence now reads "These data indicate that elevated ABA levels alone are not sufficient to explain the decrease of stomatal conductance under elevated VPD conditions, as discussed before (Wang et al., 2017; Merilo et al., 2018; Yaaran et al., 2019).", now in lines 420-423.

- lines 437-438: "changes in atmospheric VPD and CO2 levels are perhaps the greatest agricultural challenges of the future" What about drought stress?

We have revised the sentence to read "changes in atmospheric VPD and CO_2 levels are among the greatest agricultural challenges of the future" (line 447) to indicate that there are other challenges.

12-May-2024
Dr. Hanna Hõrak
Tartu Ulikool
Institute of Technology
Tartu 50411
Estonia

MSID: PP2023-RA-01350R2
MS TITLE: Stomatal CO2-responses at sub- and above-ambient CO2 levels employ different pathways in Arabidopsis

Dear Dr. Hanna Hõrak:

I am pleased to inform you that your revised manuscript "Stomatal CO2-responses at sub- and above-ambient CO2 levels employ different pathways in Arabidopsis" has been accepted for publication in Plant Physiology. Your manuscript will be checked for consistency with journal formatting and image standards by one of our Science Editors, who will contact you shortly. They may have a number of queries and suggestions for you. Please keep in mind that they work to increase the online visibility of your article and its attraction for readers from the widest of backgrounds. You will have the opportunity to review any revisions, and I hope you will work constructively with them to get the most out of your research. You will also have the opportunity to review all changes when you receive the author proofs for your article.

Once validated for publication, your article will be sent to a Science Editor for a final review of your manuscript and source files. After your final files are uploaded, your article will appear in the next available issue and the online Preview version of your article will be posted after your signed Copyright and Checklist forms and final source files are received.

Please be sure to include the following in your manuscript file:
Harvard style citations
List of all author contributions and funding information at the beginning of manuscript after the titles and author list.
List all supplemental materials associated with the article, with titles, at the end of the ms, before the references and acknowledgments section.
Complete Figure Legends for all figures appearing in the article listed before the Literature Cited.

Thank you and congratulations on your Plant Physiology paper!

Sincerely,
Editor
Plant Physiology

=====
FIRST AUTHOR PROFILES
=====

We recognize the hard work that goes into being First Author of a Plant Physiology paper by publishing a "First Author Feature" for each First Author alongside your paper.

If the first author(s) would like to submit information for a feature on Facebook and Twitter, fill out the form by clicking on the link below:

Plant Physiology First Author Feature Form: https://forms.office.com/r/ZBCGZfhm8F
If you cannot access the form through the link above, please contact Rachel at rbelsky@aspb.org.

To upload an image of yourself to accompany your profile, please put your photo in the correct journal folder at the link below. You must label your image: Author First Name Last Name Article Title.

First Author Profile Image Dropbox: https://www.dropbox.com/scl/fo/xz029p7qjr2jml2rp2kmq/h?dl=0&rlkey=6fkxcaqmxx35ap152b2osy1fz

=====
IMPORTANT REMINDER: PEER REVIEW REPORTS
=====

If you opted to publish a peer review report along with your article during the original submission process, it will be prepared by the editorial staff and publicly posted with your manuscript, inside the zip file that contains any other supplemental material. As a reminder, the peer review report is a public record of all comments from editors and reviewers, as well as your prior responses, as you received them in the decision letters for each draft of your manuscript. If you agreed to publish this report and have changed your mind, or are not sure if you selected this option, please contact the editorial office as soon as possible before signing the license agreement from our publisher.

-- Reviewer comments:
